# Reversal of tamoxifen resistance by artemisinin in ER+ breast cancer: bioinformatics analysis and experimental validation

**DOI:** 10.32604/or.2024.047257

**Published:** 2024-05-23

**Authors:** ZHILI ZHUO, DONGNI ZHANG, WENPING LU, XIAOQING WU, YONGJIA CUI, WEIXUAN ZHANG, MENGFAN ZHANG

**Affiliations:** Department of Oncology, China Academy of Chinese Medical Sciences Guang’anmen Hospital, Beijing, China

**Keywords:** Artemisinin, Tamoxifen resistance, Breast cancer

## Abstract

Breast cancer is the leading cause of cancer-related deaths in women worldwide, with Hormone Receptor (HR)+ being the predominant subtype. Tamoxifen (TAM) serves as the primary treatment for HR+ breast cancer. However, drug resistance often leads to recurrence, underscoring the need to develop new therapies to enhance patient quality of life and reduce recurrence rates. Artemisinin (ART) has demonstrated efficacy in inhibiting the growth of drug-resistant cells, positioning art as a viable option for counteracting endocrine resistance. This study explored the interaction between artemisinin and tamoxifen through a combined approach of bioinformatics analysis and experimental validation. Five characterized genes (*ar, cdkn1a, erbb2, esr1, hsp90aa1*) and seven drug-disease crossover genes (*cyp2e1, rorc, mapk10, glp1r, egfr, pgr, mgll*) were identified using WGCNA crossover analysis. Subsequent functional enrichment analyses were conducted. Our findings confirm a significant correlation between key cluster gene expression and immune cell infiltration in tamoxifen-resistant and -sensitized patients. scRNA-seq analysis revealed high expression of key cluster genes in epithelial cells, suggesting artemisinin’s specific impact on tumor cells in estrogen receptor (ER)-positive BC tissues. Molecular target docking and *in vitro* experiments with artemisinin on LCC9 cells demonstrated a reversal effect in reducing migratory and drug resistance of drug-resistant cells by modulating relevant drug resistance genes. These results indicate that artemisinin could potentially reverse tamoxifen resistance in ER-positive breast cancer.

## Introduction

Breast cancer has overtaken lung cancer as the most prevalent malignancy globally, with 2.26 million new cases annually. It is the primary cause of cancer deaths in women [1]. Eighty percent of these cases are estrogen receptor (ER)/progesterone receptor (PR) positive, making them suitable for endocrine therapy [2]. Tamoxifen, the leading drug for treating ER/PR+ breast cancer, has been clinically used for over 30 years, significantly enhancing patient prognosis and reducing breast cancer recurrence by 40% and mortality by 31% [3–6]. Nonetheless, 22%–52% of patients undergoing tamoxifen treatment still face recurrence and metastasis due to tamoxifen resistance, making the investigation of this resistance mechanism a crucial challenge in current breast cancer research [7,8]. Despite extensive studies on tamoxifen resistance signaling pathways, the complexity and partial understanding of these mechanisms continue to result in new resistance patterns [9]. While molecularly targeted agents like mTOR, CDK4/6, PIK3CA, and HDAC inhibitors are available for combination therapy in patients with HR+/HER2 metastatic cancers [10,11], their use is often limited by side effects such as gastrointestinal reactions and bone marrow suppression.

At the cellular level, breast cancer stem cells (BCSCs) with self-renewal and multidirectional differentiation potential have been identified as central to tamoxifen resistance. Aberrant activation of the Notch signaling pathway can lead to the excessive proliferation of BCSCs [12], which contributes to breast cancer development and metastasis. Furthermore, epigenetic factors such as histone modifying enzymes and non-coding RNAs are implicated in regulating the CSC phenotype. Tamoxifen-resistant cells demonstrate pronounced stem cell-like characteristics in DNA methylation and gene expression, notably Nanog, Sox, and Oct 4 [13]. Numerous studies have established that BCSCs are pivotal in acquiring tam resistance in ER+ breast cancer, serving as a central axis in tamoxifen resistance. However, these cells are not static entities; their dynamic differentiation and de-differentiation with non-stem cells render them challenging targets for specific therapies. Developing new therapeutic strategies and drugs to counteract breast cancer tamoxifen resistance is imperative.

Artemisinin (ART), a Traditional Chinese Medicine, has been widely used as an antimalarial due to its effectiveness and low toxicity [14,15]. Recent research indicates that ART inhibits the growth of various cancer types, including breast, lung, ovarian, prostate, and melanoma, including many drug-resistant cancer cell types [16–18]. Clinical use of ART has shown promising results in treating endocrine-resistant breast cancer patients [16], enhancing patient quality of life and prolonging progression-free survival. ART may represent a novel candidate for combating endocrine resistance. This study is the first to investigate the reversal of tamoxifen resistance in ER-positive BC by artemisinin, employing comprehensive bioinformatics analysis.

## Materials and Methods

### Data acquisition

The RNA sequencing data of the MCF-7 (ER-positive endocrine therapy sensitive human BC) cell line and LCC9 (dual tamoxifen and fulvestrant endocrine-resistant induced from MCF-7) cell line were obtained from the Gene Expression Omnibus (GEO, https://www.ncbi.nlm.nih.gov/geo/) database, accession number GSE159968 [19]. The expression profile by array and clinical information on distant metastasis in 298 ER-positive BC patients treated with tamoxifen for 5 years were collected from the GEO database, accession number GSE17705 [20]. The single-cell RNA sequencing data of 6 ER-positive BC patients’ tumor tissue were acquired from the GEO database, accession number GSE161529 [21]. Relative gene expression data for MCF-7 cell lines were sourced from the Cancer Cell Line Encyclopedia database (CCLE, https://sites.broadinstitute.org/ccle/) [22]. Information of datasets as shown in Table 1.

**Table 1:** Information of datasets

Dataset	Platform	Origin	Species
GSE159968	GPL20115	Invasive ductal carcinoma of breast	*Homo sapiens*
GSE17705	GPL96	Invasive ductal carcinoma of breast	*Homo sapiens*
GSE161529	GPLL18573	Invasive ductal carcinoma of breast	*Homo sapiens*

### Extraction of artemisinin targets

The chemical information and 2D structure of artemisinin (PubChem CID: 68827) was retrieved from PubChem (https://pubchem.ncbi.nlm.nih.gov/) database. Predictions of the targets of artemisinin were performed by combinatorial utilization of four databases, including CTD (http://ctdbase.org/), SwissTargetPrediction (http://www.swisstargetprediction.ch/), BindingDB (http://bindingdb.org/bind/index.jsp), and TargetNet (http://targetnet.scbdd.com/home/index/). Targets from SwissTargetPrediction and TargetNet were screened by the criterion of probability >0. The targets’ gene symbol was annotated through the STRING (https://string-db.org/) and UniProt (https://www.uniprot.org/) websites.

### Identification of tamoxifen resistance associated genes

Differentially expressed genes (DEGs) between tamoxifen-sensitive BC cells MCF-7 and tamoxifen-resistant BC cells LCC9 were identified using the limma [23] (version: 3.52.4) R package, with an adjusted *p* value < 0.05 and |logFC| > 1. The ER-positive BC patients (n = 298) were classified based on distant metastasis. Recurrence within 3 years was considered as resistance, while no recurrence after >10 years indicated sensitivity. Differential expression analysis among resistant and sensitive patients was performed using the limma package. The top 5,000 DEGs (based on *p* value) were selected for Weighted Correlation Network Analysis (WGCNA), employing the WGCNA [24] R package. A scale-free R^2^ = 0.9 was chosen, with soft-threshold parameters β ranging from 1 to 30 [25]. The optimal β value was determined using the function “sft$powerEstimate”. A cluster dendrogram was generated based on the topological overlap matrix, with a minimum cluster size of 50. Genes with similar expression profiles were grouped into modules, and those with a dissimilarity <0.25 were merged. Module–trait relationships analysis was conducted to identify modules involved in tamoxifen resistance. Modules with a correlation coefficient > 0.3 and *p* < 0.05 were deemed significant. The intersection of DEGs between MCF-7 and LCC9 and significant module genes from WGCNA were hypothesized to be tamoxifen resistance-associated genes (TAMGs).

### DO, KEGG, GSEA and CIBERSORT analysis

The Kyoto Encyclopedia of Genes and Genomes (KEGG) enrichment analysis, Disease Ontology (DO) enrichment analysis, and Gene Set Enrichment Analysis (GSEA) were performed using the clusterProfiler [26] (version: 4.4.4), DOSE [27] (version: 3.22.1), GSEABase (version: 1.58.0), and GSVA [28] (version: 1.44.5) R packages. Filter criteria were set to *p* < 0.05 and false discovery rate (FDR) <0.05. The abundance of 22 immune cells in each sample was assessed using the IOBR [29] R package with the CIBERSORT method [30]. The difference in immune cell proportions between groups was analyzed using the Mann-Whitney signed-rank test. Spearman correlation analysis was employed to explore relationships between specific gene expression levels and immune cell infiltration, with *p* value < 0.05 considered significant.

### MCODE analysis

The gene list, comprising artemisinin target genes and TAMGs, was uploaded to the STRING database (https://string-db.org/) to create a protein-protein interaction (PPI) network. Interactions with combined scores below 0.4 and disconnected nodes were excluded. The refined network was imported into Cytoscape (version: 3.7.2) software, and subnetworks were generated using the MCODE (version: 1.6.1) plugin. Subnetworks with scores above 5 were selected as key cluster candidates.

### scRNA-seq analysis

The Seurat (v4.0.6) R package processed scRNA-seq data from 6 ER-positive BC tissues [31]. Quality control parameters were set as follows: genes expressed in >3 cells, UMI count ≥ 1000, cells expressing 200–10,000 genes, and ≤20% mitochondrial and ribosomal counts. The Seurat object was normalized using LogNormalize, and batch effects were corrected with RunHarmony. The top 2000 variable genes for dimensionality reduction were identified by FindVariableFeatures. Dimheatmap, JackStrawPlot, and ElbowPlot were utilized to determine principal components (PCs) and clustering resolution. TSNE (T-distributed Stochastic Neighbor Embedding) visualized dimensional reduction. Marker genes for each cluster were identified using FindAllMarkers. The SingleR (version: 1.8.0) package classified cell clusters using the reference loaded from the celldex (version: 1.4.0) package via the HumanPrimaryCellAtlasData function. Enrichment scores were calculated using the irGSEA (v1.1.2) R package with the “AUCell,” “UCell,” and “singscore” algorithms.

### Component-target molecular docking

The 2D structure of the ligand artemisinin was obtained from PubChem (https://pubchem.ncbi.nlm.nih.gov/) (CID: 68827). The Protein Data Bank (PDB) entry IDs of receptor proteins were sourced from the STRING database, and their corresponding crystal structures were downloaded from the PDB database (https://www.rcsb.org/). Using AutoDock Tools (version: 1.5.6), water molecules were removed, proteins isolated, nonpolar hydrogens added, and structures saved as PDBQT files. Semi-flexible docking was conducted with AutoDock Vina [32] (version: 1.2.0), treating receptors as rigid and ligands as flexible. Twenty conformations were generated for each compound. Docking results were analyzed and visualized using Discovery Studio (version: 4.5).

## Cell Culture

### Cell lines and cell culture

MCF7R/LCC9 cells, presented by Professor Clarke of Georgetown University Medical Center, and MCF7 cells, acquired from the Cell Center, Chinese Academy of Medical Sciences, were cultured in DMEM medium (Gibco, New York, USA) supplemented with 10% fetal bovine serum (FBS, Gibco, New York, USA), 1% penicillin-streptomycin (Gibco, New York, USA), and 10% insulin (Gibco, New York, USA) in a 5% CO_2_ humidified atmosphere at 37°C. Additionally, LCC9 cells were cultured with 1% 4-OH tamoxifen (Sigma-Aldrich, St. Louis, Missouri, USA).

### Cell viability assay

Cells (MCF-7: 6000 cells/well, LCC9: 8000 cells/well) were seeded in a 96-well plate and incubated for 24 h. They were treated with various concentrations (0–30 µM) of 4-OH tamoxifen for 24 or 48 h, followed by treatment with different concentrations (0–30 µM) of art (Genye Biologics, Shanghai, China) for 24 or 48 h. The cytotoxic effects of tam and art on LCC9 cells, and the effect of tam on sensitive MCF-7 cells, were evaluated using the CCK8 assay. Subsequently, CCK-8 solution (Dojindo Laboratories, Kyushu, Japan) was added and incubated for 2 h in a CO_2_ incubator. The absorbance of the final solution was measured at 450 nm using an ELx808 Automatic Microplate Reader (Biotek Corporation of America, USA).

$$\;\begin{array}{l} \;Cell\;Viability\;(\%\;of\;control)\;=\\ \;\frac{absorption\;(treated\;cells)-absorption\;(medium\;alone)}{absorption\;\left(untreated\;cells\right)-absorption\;\left(medium\;alone\right)}*100 \end{array}$$

### Transwell experiment

Cells were exposed to media containing 1% FBS, with interventions including art, tam, or a combination of both. Approximately 1 × 10^5^ cells in 200 µL of medium were placed in the upper chamber of transwell inserts (Corning Company, NY, USA). The lower chamber was filled with complete medium containing 20% FBS. The cells were then incubated at 37°C for 48 h to allow migration. At designated time points, images of the migrated cells were captured and analyzed using ImageJ software (version 1.48v, NIH).

### Total RNA extraction and real-time polymerase chain reaction (RT-PCR)

Following 48-h treatment with art, tam, or their combination, total RNA was isolated using Trizol reagent (Thermo Fisher Scientific, Massachusetts, USA). Reverse transcription was performed using a cDNA kit (primer sequences in Table 2). Quantitative real-time PCR assessed the mRNA levels of specific genes. The 2^−ΔΔCT^ method calculated the fold change, normalizing the results to the internal control (ACTIN), where ΔΔCT = ΔCT(a target sample) − ΔCT(a reference sample), ΔCT = CT(a target gene) − CT(a reference gene).

**Table 2:** The primer sequences

Gene	Forward	Reverse
*actin*	5′-TGTCCACCTTCCAGCAGATGT-3′	5′-GCTCAGTAACAGTCCGCCTAGA-3′
*ar*	5′-CTACATCAAGGAACTCGATCGT-3′	5′-CATGTGTGACTTGATTAGCAGG-3′
*cdkn1a*	5′-GATGGAACTTCGACTTGTCAC-3′	5′-GTCCACATGGTCTTCCTCTG-3′
*erbb2*	5′-CCAGCTCTTTGAGGACAACTAT-3′	5′-TTTCAAGATCTCTGTGAGGCTT-3′
*esr1*	5′-TACTGCATCAGATCCAAGGGAA-3′	5′-CCTCGGGGTAGTTGTACAC-3′
*hsp90aa1*	5′-CCAGTTCGGTGTTGGTTTTTAT-3′	5′-CAGTTTGGTCTTCTTTCAGGTG-3′
*pgr*	5′-CGTACCCTCTCTATAGCGACTT-3′	5′-ACCGGCCACAAGGTAGGAA-3′
*egfr*	5′-ACCCATATGTACCATCGATGTC-3′	5′-GAATTCGATGATCAACTCACGG-3′
*cyp2e1*	5′-CCATCAAGGATAGGCAAGAGAT-3′	5′-ATTCAGGAAGTGTTCTGGCTTA-3′
*glp1r*	5′-TCTCTGCTCTGGTTATCGCCTCTG-3′	5′-ACAATGCTCGCAGGATGAAGGATG-3′
*mapk10*	5′-GAGCAAAAGCAAAGTTGACAAC-3′	5′-TAGGCTTTAGATTCTGGTAGCG-3′
*mgll*	5′-TAGTGTCTGACTTCCACGTTTT-3′	5′-GAACCAGAGGCGAAATGAGTA-3′
*rorc*	5′-AGTAGAACAGCTGCAGTACAAT-3′	5′-CTGAAGAGCTCCTTGTAGAGTG-3′

### Western blot (WB)

Total intracellular proteins were extracted using RIPA lysis buffer (Beyotime, Shanghai, China), with the addition of a protease inhibitor mixture (Beyotime, Shanghai, China) and 0.1 mM PMSF (Beyotime, Shanghai, China). Protein concentration was measured using a BCA assay kit (Beyotime, Shanghai, China). Subsequent steps included protein denaturation, electrophoresis, and membrane transfer as per the manufacturer’s guidelines. Procedures for milk blocking, membrane washing, and overnight primary antibody incubation followed. After 2 h of secondary antibody incubation, target proteins were detected using the Beyo ECL Star Kit (Beyotime, Shanghai, China) and quantified with Bio-Rad Quantity One software. Information about protein blotting antibodies is in Table 3.

**Table 3:** Western blot antibody materials

Products	Factory	Cat. no.
Protein extract	MDL, Hebei, China	MDL91201
Protease inhibitor	MDL, Hebei, China	MD912893
BCA protein concentration measurement kit	MDL, Hebei, China	MD913053
SDS-PAGE preformed gel kit	MDL, Hebei, China	MD911919
Protein secondary antibody	MDL, Hebei, China	MD912565
Protein lysate	Genye Biologics, Shanghai, China	P0013
Medium protein molecular weight marker	Genye Biologics, Shanghai, China	P0080
Actin antibody	Affinity, Jiangsu, China	AF7018
HSP90AA1 antibody	Affinity, Jiangsu, China	BF0084
ESR1 antibody	Affinity, Jiangsu, China	BF0200
AR antibody	Affinity, Jiangsu, China	AF6137
ERBB2 antibody	Affinity, Jiangsu, China	AF7681
CDKN1A antibody	Affinity, Jiangsu, China	AF6290

### Statistical analysis

Data are presented as mean ± SD from three independent experiments and analyzed using one-way analysis of variance (ANOVA) with IBM SPSS Statistics 27.0 software (SPSS, USA). Differences with a *p* value < 0.05 were considered significant.

## Results

### Artemisinin has a potential therapeutic effect on ER positive BC

**Figure 1:**
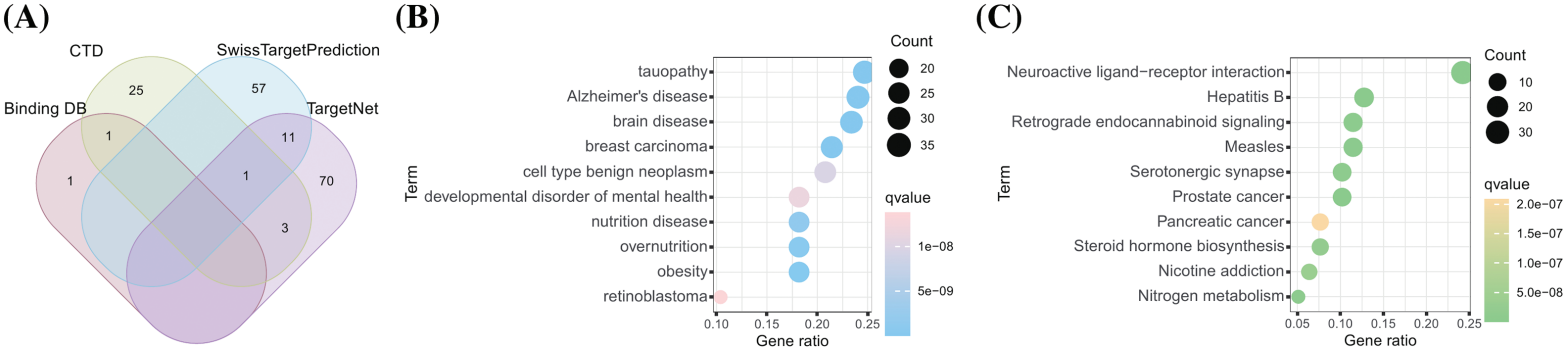
A total of 169 drug-targets were retrieved from 4 databases. (A) The venn gram of 169 artemisinin targets predicted from 4 databases; (B) DO analysis burble plot of artemisinin targets; (C) KEGG enrichment analysis burble plot of artemisinin targets.

A total of 169 artemisinin target genes were identified from 4 databases (Fig. 1A). DO enrichment analysis indicated significant enrichment of these genes in 391 different diseases (Suppl. Table S1). The top 10 significant DO terms, arranged by gene ratio, included breast carcinoma, mental health disorders, nutrition diseases, overnutrition, and obesity (Fig. 1B). The top 10 significant KEGG terms by gene proportion are shown in (Fig. 1C). Notably, robust evidence suggests a link between nutritional metabolic disorders, obesity, and the progression of tumors, particularly ER-positive BC in postmenopausal women [33,34]. Additionally, mental illness has been reported to significantly impact BC mortality [35]. KEGG pathway enrichment analysis of these target genes showed significant enrichment in 132 pathways (Suppl. Table S2). Notably, among the top 10 enriched KEGG terms was steroid hormone biosynthesis; estrogen, a steroid hormone, plays a crucial role in ER-positive BC.

### Acquisition of TAMGs from clinical patients and cell lines

**Figure 2:**
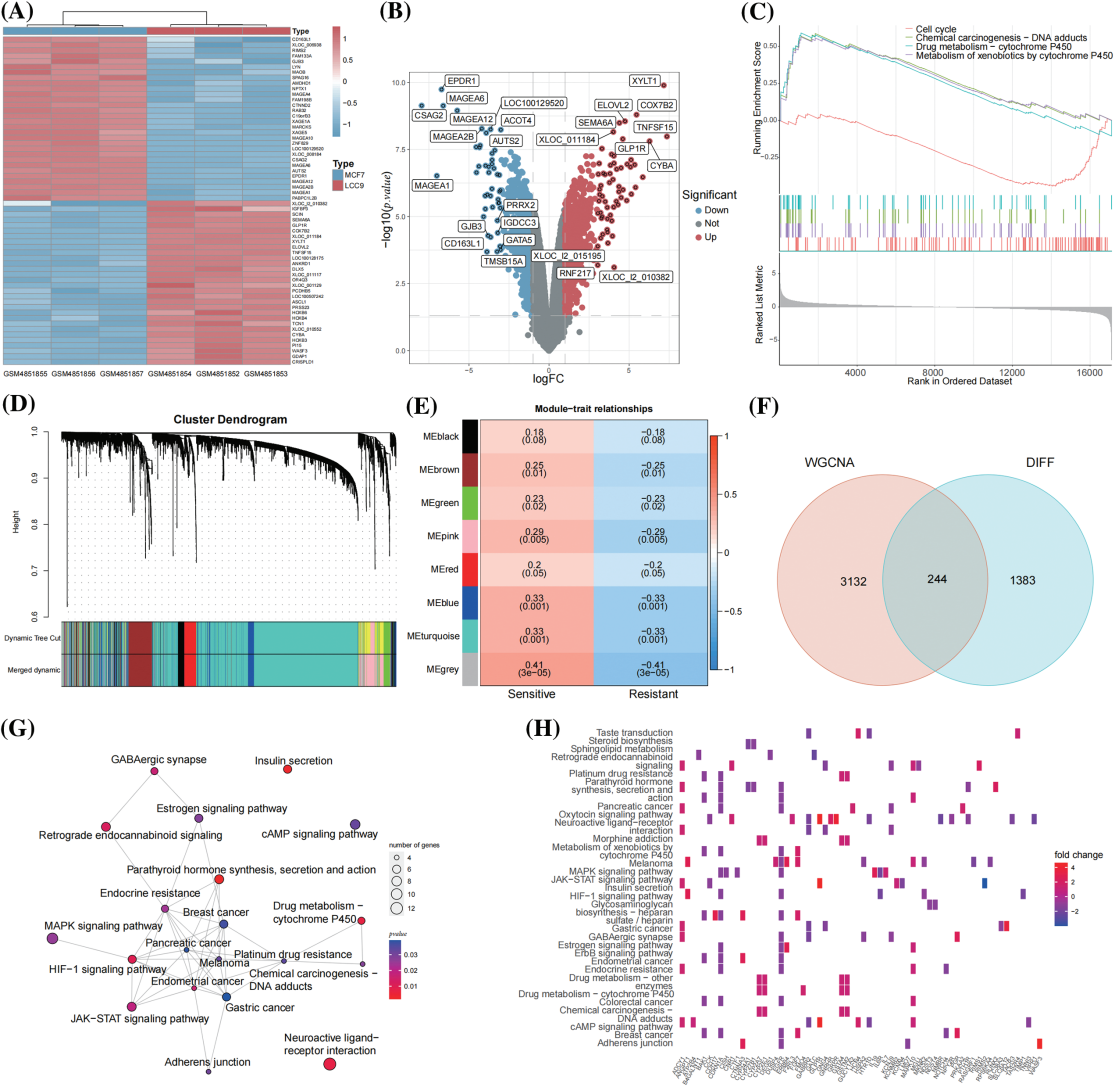
A total of 244 TAMGs were acquired from BC cell lines and clinical patients. (A) Heatmap of the top 60 DEGs between MCF-7 and LCC9 cell lines; (B) Volcano plot labeled the DEGs with |logFC| > 3; (C) GSEA analysis of the DEGs; (D) Gene dendrogram and module colors; (E) The heatmap of module-trait relationships; (F) Venn map of the WGCNA module genes and DEGs; (G) The network of KEGG pathways enriched by 244 TAMGs; (H) The heatmap of TAMGs within the enriched pathways.

Differential expression analysis between the endocrine-sensitive cell line MCF-7 and the endocrine-resistant cell line LCC9 identified 1627 DEGs. The top 60 DEGs are depicted in a heatmap (Fig. 2A), with gene symbols of DEGs having |logFC| > 3 highlighted in a volcano plot (Fig. 2B). Gene Set Enrichment Analysis (GSEA) of these DEGs showed significant enrichment in cell cycle and drug metabolism-cytochrome P450 pathways (Fig. 2C). Large clinical trials have indicated that poor cytochrome P450 metabolizers are likely to have a compromised response to tamoxifen [36]. Dysregulation in cell-cycle machinery is a key characteristic in tumorigenesis, observed in various cancer types [37].

From the GSE17705 dataset (298 ER+ BC patients), 19 were identified as tamoxifen-resistant relapses (<3 years), and 77 as tamoxifen-sensitive (≥10 years, no metastasis). WGCNA analysis was conducted on the expression matrix of 96 patients with tamoxifen response as clinical traits. The optimal soft threshold value was determined to be 4 using the function “sft$powerEstimate”. The one-step network construction function identified modules (Fig. 2D) and established module-trait relationships (Fig. 2E). The top 5000 differentially expressed genes were categorized into 8 modules, with 85 genes not co-expressed, forming the grey module, which is not considered a real module. Correlation coefficients between modules and clinical traits revealed that the blue and turquoise modules were significantly associated with tamoxifen response (correlation coefficient > 0.3 and *p* < 0.05). Consequently, 470 genes from the blue module and 2906 from the turquoise module were extracted and merged for further analysis (Suppl. Table S3). The intersected 244 genes from 3376 WGCNA module genes and 1627 DEGs were identified as TAMGs (Fig. 2F). These 244 TAMGs underwent KEGG pathway enrichment analysis (Fig. 2G). The KEGG pathway analysis revealed pathways related to various cancers, cancer signaling, and drug responses, including endocrine resistance. These findings suggest that the 244 TAMGs identified in this study are likely pivotal direct genes in ER-positive tamoxifen-resistant BC. TAMGs within the enriched pathways are visualized in a heatmap (Fig. 2H).

### Acquisition of key cluster genes of artemisinin in the treatment of tamoxifen resistant BC

**Figure 3:**
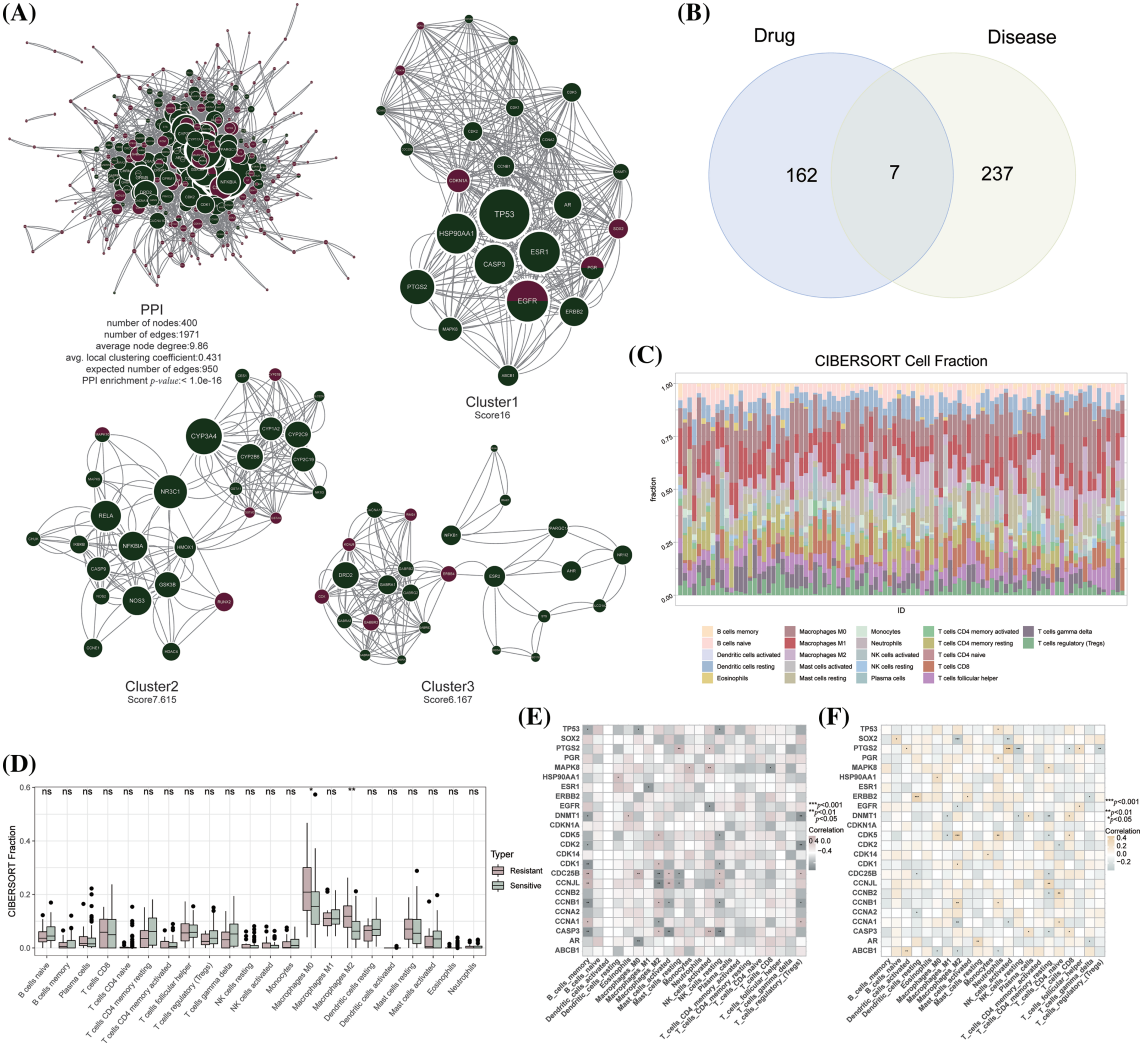
Acquisition of key cluster genes of artemisinin in the treatment of tamoxifen resistant BC. (A) The PPI network and sub-clusters of artemisinin targets and TAMGs; (B) The venn gram of 169 artemisinin targets and 244 TAMGs; (C) The barplot of 22 immune cells fraction in sensitive and resistant patients by using CIBERSORT method; (D) The boxplot of differences in immune cells fraction between resistant and sensitive patients (ns: non-significant, *p < 0.05, **p < 0.01, ***p < 0.001); (E) The heatmap of correlation between key-cluster genes and immune cells fraction in resistant patients; (F) The heatmap of correlation between key-cluster genes and immune cells fraction in sensitive patients.

The PPI network of these gene sets was constructed, and 3 sub-clusters with scores above 5 were isolated using the MCODE algorithm (Fig. 3A). Seven genes were identified as common between the 244 TAMGs and 169 artemisinin target genes, including *cyp2e1, rorc, mapk10, glp1r, egfr, pgr, and mgll* (Fig. 3B). Red nodes represent TAMGs, green nodes denote artemisinin targets, and larger node sizes indicate higher degree values. The figure reveals that artemisinin’s function extends beyond the intersection genes, as non-intersecting target genes also significantly interact with TAMGs. Among the 3 sub-clusters, cluster 1 contains 25 nodes and 384 edges, cluster 2 has 27 nodes and 198 edges, and cluster 3 comprises 25 nodes and 148 edges, making cluster 1 the top priority as a key cluster. The immune infiltration scores of 19 resistant and 77 sensitive patients were calculated using the CIBERSORT algorithm (Fig. 3C). Among 22 immune cell types, the proportions of macrophages M0 and M2 were notably higher in resistant patients compared to sensitive patients (Fig. 3D). This finding aligns with expectations, as M2 macrophages, known for their anti-inflammatory effects, promote cancer growth and invasion [38]. Tumor-associated macrophages are associated with tamoxifen resistance in postmenopausal breast cancer patients [39], study shows that sodium/glucose cotransporter 1 (SGLT1) overexpression drives the highly glycolytic phenotype of tamoxifen-resistant breast cancer cells where enhanced lactic acid secretion promotes M2-like tumor-associated macrophages polarization via the hypoxia-inducible factor-1α/signal transducer and activator of transcription-3 pathway [40]. Correlation analysis between the expression levels of key cluster genes and immune cell infiltration was conducted separately in tamoxifen-resistant and -sensitive patients (Figs. 3E, 3F). The results demonstrated that key cluster genes significantly correlate with different types of immune cells in both patient groups, suggesting that these genes might play dual roles in the tumor microenvironments of resistant and sensitive patients.

### scRNA-seq analysis showed that BC cells were sensitive to artemisinin

**Figure 4:**
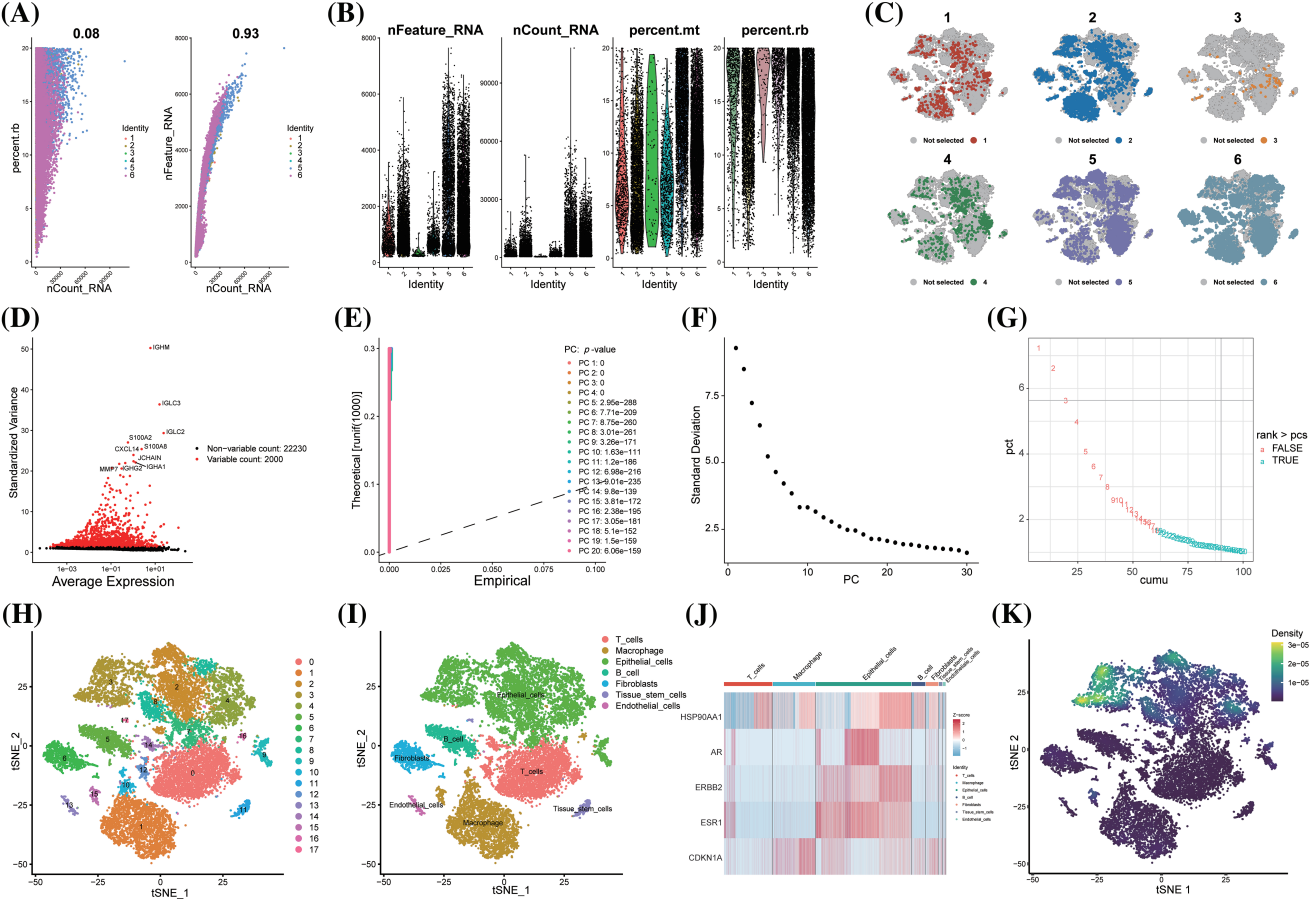
scRNA-seq analysis showed that BC cells were sensitive to artemisinin. (A) The scatter plots of the correlation between sequencing depth and ribosomal RNA percentage and mitochondrial genes; (B) The violin plots of the identities of 6 ER-positive BC samples; (C) The TSNE distribution maps of 6 ER-positive BC samples after batch effect removal; (D) The volcano plot of the top 2000 highly variable genes, the top 10 genes were marked out; (E) The *p*-values of 1-20 PCs computed through JackStraw function; (F) The ElbowPlot of standard deviation from 1 to 30 PCs; (G) The cumulative percentages for 1 to 30 PCs, 18 is the last point with a change of variation percentage more than 0.1%; (H) The tSNE algorithm divided the cells from 6 ER-positive BC samples into 18 clusters; (I) The 18 clusters were annotated into 7 cell types; (J) The heatmap of 5 signature genes selected from key-cluster genes in 7 cell types; (K) The prediction of artemisinin response in single cells via the expression level of key-cluster genes, the epithelial cells demonstrated a significantly higher sensitivity.

Post-quality control, Pearson’s correlation analysis was conducted between sequencing depth and both ribosomal RNA percentage and mitochondrial genes; the *p* values exceeded 0.05, indicating no significant correlation (Fig. 4A). The identities of scRNA-seq cells used in downstream analysis were characterized, including sequencing depth, gene numbers, mitochondrial RNA percentage, and ribosomal RNA percentage (Fig. 4B). Following batch effect removal (Fig. 4C), the top 2000 highly variable genes across cells were identified (Fig. 4D). The principal components (PCs) value was determined using three methods: dimheatmap (Suppl. Fig. S1), JackStraw function (Fig. 4E), and ElbowPlot function (Fig. 4F). In the elbow plot, the absence of a clear elbow point and all *p* values calculated by JackStraw being <0.01 led to further computation of cumulative percentages for each PC (Fig. 4G). A value of 18 emerged as the optimal PC, as it represents the last point where the change in percentage variation exceeds 0.1%. The resolution was set at 0.5 via clustree (Suppl. Fig. S2). TSNE (T-distributed Stochastic Neighbor Embedding) dimensionality reduction visualized 18 clusters of single cells (Fig. 4H), with the top 5 significant marker genes (ar, cdkn1a, erbb2, esr1, hsp90aa1) of each cluster shown in a heatmap (Suppl. Fig. S3). These 18 cell clusters were annotated into 7 cell types (T cells, B cells, epithelial cells, fibroblasts, tissue stem cells, endothelial cells and macrophage) (Fig. 4I, Suppl. Fig. S4). The irGSEA package estimated the expression level of key-cluster genes in these single cells, identifying 5 genes as signature genes due to their highly variable expression levels across cell types (Fig. 4J). The irGSEA score for each cell, based on the expression levels of key-cluster genes, revealed that epithelial cells had significantly higher levels of these genes (Fig. 4K). In BC tissues, epithelial cells predominantly comprise tumor cells. The specific high expression of these key-cluster genes in epithelial cells implies that artemisinin exerts a considerable specific effect on tumor cells in ER-positive BC tissue.

### Molecular docking of artemisinin between 5 signature genes and 7 drug-disease intersect genes

**Figure 5:**
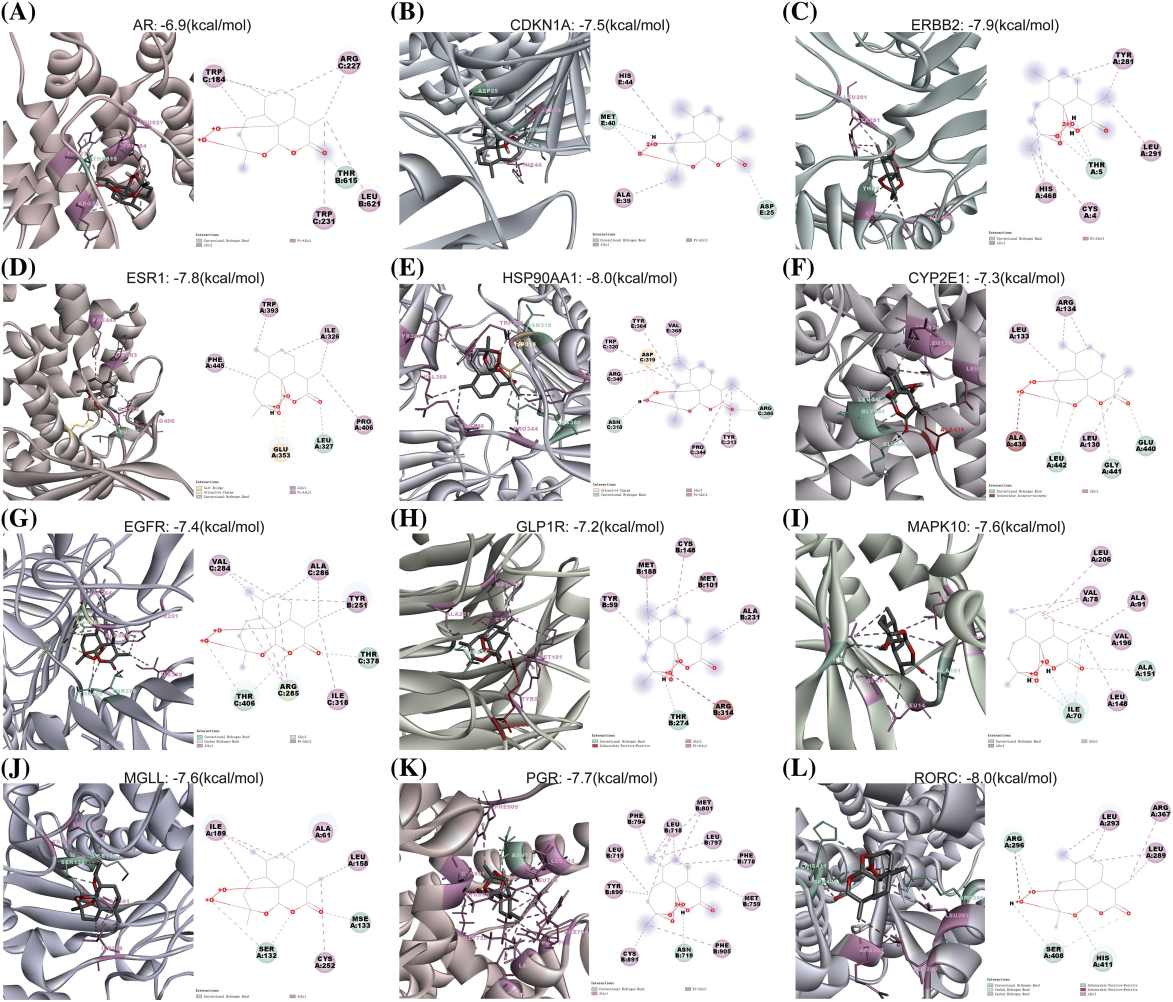
The 3 and 2d molecular docking conformation diagram. (A–L) The docking conformation of the *ar, cdkn1a, erbb2, esr1, hsp90aa1, cyp2e1, egfr, glp1r, mapk10, mgll, pgr* and *rorc* molecule, respectively. The green, light green, pink, yellow and red broken lines represent the conventional hydrogen-bonding, carbon hydrogen bond, alkyl/pi-alkyl, attractive charge and unfavorable acceptor-acceptor/positive-positive interactions, respectively.

Molecular docking experiments were conducted using AutoDock Vina software to simulate and confirm the interactions between the small-molecule ligand artemisinin and large-molecule target proteins. This process involved the 5 characteristic genes (*ar, cdkn1a, erbb2, esr1, hsp90aa1*) and seven drug-disease crossover genes (*cyp2e1, rorc, mapk10, glp1r, egfr, pgr, mgll*), each undergoing molecular docking with artemisinin (Figs. 5A–5L). We evaluated the molecules of five characterized genes and seven drug-disease crossover genes with artemisinin on the basis of binding energy (docking score) for the docking results, as shown in Table 4. The results indicated that all 12 proteins exhibited strong affinity (docking score <−5 kcal/mol) with artemisinin and formed hydrogen bonds. This suggests that artemisinin may act directly on tamoxifen-resistant ER-positive BC by binding with these target proteins.

**Table 4:** Results of molecular docking of five characterized genes and seven drug-disease cross-cutting genes with artemisinin

Protein	PDB ID	Docking score (kcal/mol)
Androgen Receptor (*ar*)	3btr	−6.9
Cyclin Dependent Kinase Inhibitor 1A (*cdkn1a*)	2zvw	−7.5
Erb-B2 Receptor Tyrosine Kinase 2 (*erbb2*)	3wlw	−7.9
Estrogen Receptor 1 (*esr1*)	2ocf	−7.8
Heat Shock Protein 90 Alpha Family Class A Member 1 (*hsp90aa1*)	3q6n	−8.0
Cytochrome P450 Family 2 Subfamily E Member 1 (*cyp2e1*)	3t3z	−7.3
Epidermal Growth Factor Receptor (egfr)	5wb7	−7.4
Glucagon Like Peptide 1 Receptor (*glp1r*)	6b3j	−7.2
Mitogen-Activated Protein Kinase 10 (*mapk10*)	3ttj	−7.6
Monoglyceride Lipase (*mgll*)	3jw8	−7.6
Progesterone Receptor (*pgr*)	1sqn	−7.7
Retinoic Acid-Related Orphan Receptor C (*rorc*)	5ntw	−8.0

### In vitro experiments indicate the reversal effect of artemisinin on tamoxifen resistance

**Figure 6:**
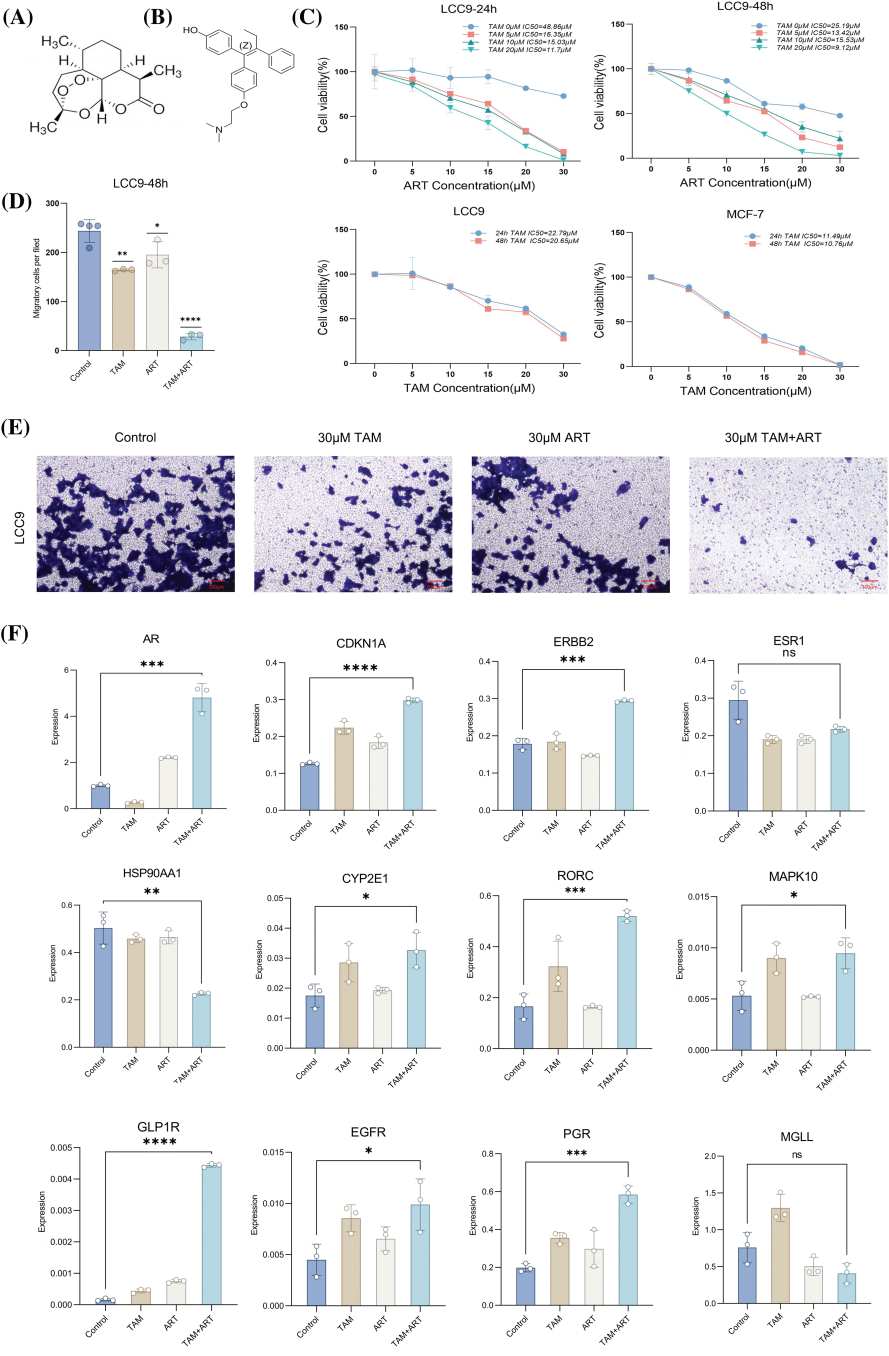
*In vitro* CCK8 assay, Transwell assay and PCR assay validation. Chemical structure of art. (A) Chemical structure of 4-OH tam. (B) Cell suppression rate of MCF-7 or LCC9 cells treated with art or tam for 24–48 h was determined by CCK-8 method. (C) Histogram of migratory capacity of four groups of LCC9 cells after treatment with art or tam for 48 h. (D) Migratory capacity of four groups of LCC9 cells after treatment with art or tam for 48 h. (E) Relative expression levels of 5 characteristic genes (*ar, cdkn1a, erbb2, esr1, hsp90aa1*) and seven drug-disease crossover genes (*cyp2e1, rorc, mapk10, glp1r, egfr, pgr, mgll*) in LCC9 cells treated with different concentrations of art or tam or a combination for 48 h. Scale bar = 100 μm. (F) These experiments are repeated at least three times. ns: not significant, *p < 0.05, **p < 0.01, ***p < 0.001, ****p < 0.0001.

The chemical structures of artemisinin and 4-ohtam are shown in (Figs. 6A, 6B). The CCK8 assay results showed that combined treatment of tam and art decreased the viability of LCC9 cells in a dose-dependent and time-dependent manner. This combination treatment had a more potent antitumor effect compared to art or tam alone. After 48 h, the IC50 value of art-treated LCC9 cells for tam was similar to that of sensitive MCF-7 cells, suggesting that art can reduce LCC9 cells’ resistance to tamoxifen (Fig. 6C). Transwell assays to assess the impact of art and tam on LCC9 cell migration revealed that the control group had 240.33 ± 27.21 migrated cells, the 30 μM tam group had 164.33 ± 2.08, and the 30 μM art group had 195.33 ± 26.63. The combined treatment significantly reduced cell migration, with only 28.67 ± 6.66 migrated cells (Figs. 6D, 6E).

**Figure 7:**
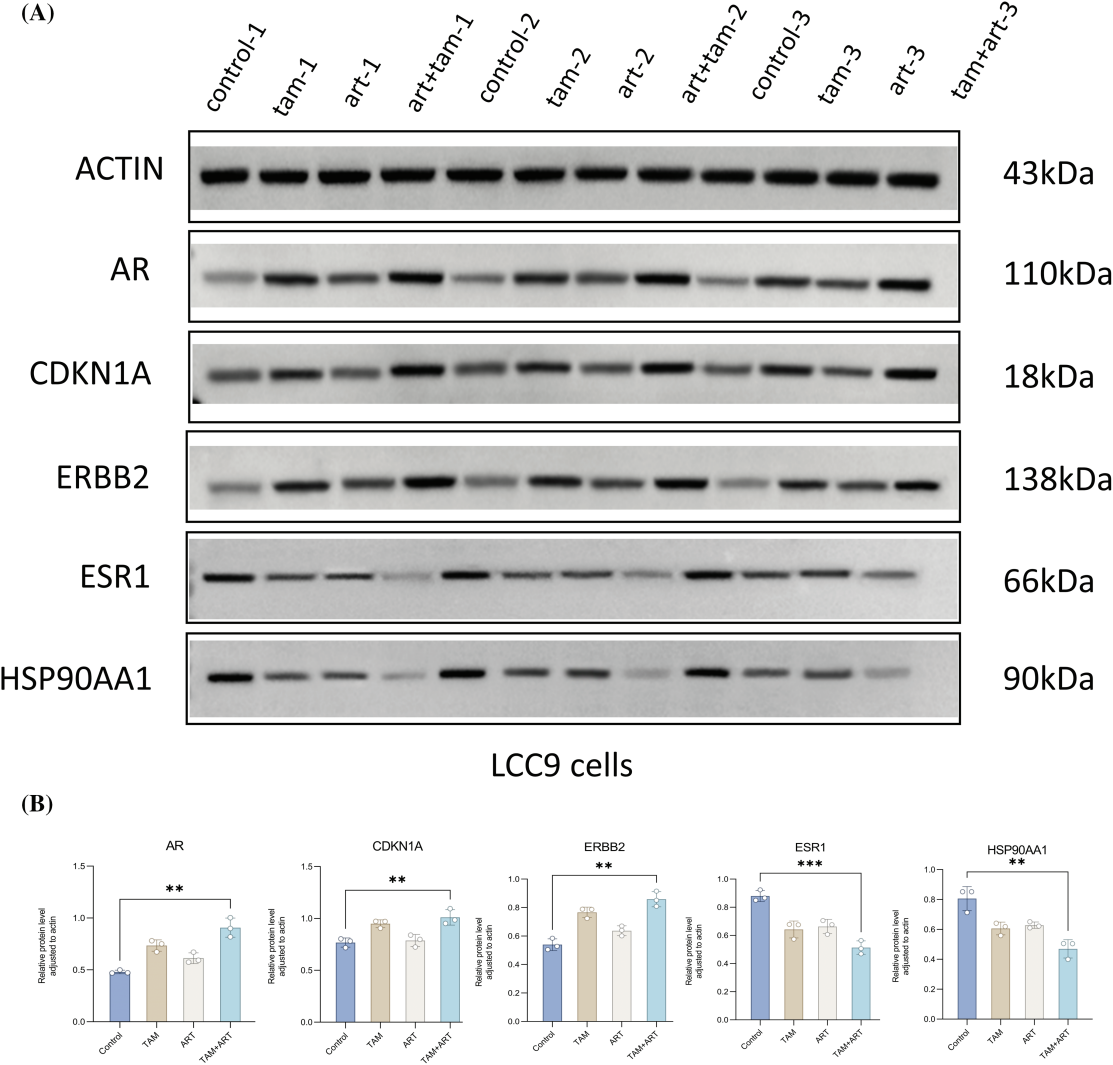
The protein expression levels of five characteristic genes (*ar, cdkn1a, erbb2, esr1, hsp90aa1*) in LCC9 cells after 48 h of intervention. (A and B) These experiments are repeated at least three times. **p < 0.01, ***p < 0.001.

RT-PCR assessed mRNA expression levels of five characteristic genes and seven drug-disease crossover genes in LCC9 cells (Fig. 6F). Comparison with MCF-7 gene expression in sensitive cells (Suppl. Table S4) showed that only *ar* gene expression was similar to MCF-7 post co-intervention in LCC9. While other genes’ expression varied significantly from MCF-7, the overall trend aligned with expectations. The Western blot experiment confirmed changes in the protein levels of five key target genes after a 48-h intervention in LCC9 cells (Figs. 7A, 7B), with results consistent with PCR trends. These experiments preliminarily demonstrated artemisinin’s potential to reverse TAM resistance in LCC9 cells.

## Discussion

In this study, we presented an integrative bioinformatics analysis to explore the reversal effect of artemisinin on tamoxifen resistance in ER-positive BC for the first time. Additionally, the role of artemisinin in enhancing the sensitivity of endocrine-resistant LCC9 cells to tamoxifen was preliminarily validated through molecular docking and *in vitro* experiments.

Artemisinin has previously demonstrated an excellent safety and tolerability profile in cancer therapy [41]. The results from DO and KEGG enrichment analyses suggest that artemisinin target genes are both directly and indirectly associated with ER-positive BC, indicating its therapeutic potential. It is been reported that hybridizing artemisinin with estrogens can potentiate their anticancer activities, creating synergistic effects among connected pharmacophores [42]. However, the involvement of artemisinin in the therapy of tamoxifen-resistant ER-positive BC had not been explored before this study.

For the identification of pronounced co-expression genes contributing to tamoxifen sensitivity differences between patients, those relapsing within 3 years were classified as resistant, while those without recurrences for over 10 years were deemed sensitive. Typically, primary endocrine resistance in breast cancer is defined as recurrence within 2 years. This filtration and classification method amplified differences between groups but may have also missed some functional gene associations during WGCNA analysis. This represents a potential bias and limitation of this study.

TAMGs were identified through differential expression analysis and WGCNA. KEGG pathway analysis highlighted enrichment in BC, cancer signaling, endocrine resistance, and drug metabolism cytochrome P450. Similarly, GSEA of DEGs between LCC9 and MCF-7 cells also indicated enrichment in drug metabolism cytochrome P450. Cytochrome P450 enzymes play a crucial role in drug metabolism and influence tamoxifen response. Previous research has shown that cytochrome P450s can impact the efficacy of anticancer agents, including tamoxifen, by mediating their biotransformation [43]. These findings corroborate with existing literature, affirming the validity of the TAMGs identified in this study.

The overlap between artemisinin target genes and TAMGs was modest, with only 7 shared genes. Nevertheless, these genes are pivotal in cancer development and progression. Cytochrome P450 family 2 subfamily E member 1 (*cyp2e1*) is linked to insulin resistance and oxidative stress, contributing to non-alcoholic fatty liver disease [44], a condition with established associations to increased BC risk [45]. Glucagon-like peptide 1 receptor (*glp1r*), the receptor for glp1, sees elevated expression following diabetes treatment with GLP1 and its analogs, potentially promoting BC malignancy [46,47]. Epidermal growth factor receptor (*egfr*), a member of the *erbb* family, plays a critical role in tumorigenesis. Mutations in *egfr* are therapeutic targets for specific antibodies and vaccines [48], and the EGFR/PI3K/AKT signaling pathway is implicated in breast carcinogenesis [49,50]. Mitogen-activated protein kinase 10 (*mapk10*), part of the *mapk* family, is involved in over forty percent of human cancer cases due to the hyperactivation of the MAPK signaling pathway, a well-established pathway in cancer biology [51]. The progesterone receptor (*pgr*) is essential in BC as a biomarker for predicting endocrine therapy response [52]. Monoglyceride lipase (MGL) regulates physiological and pathophysiological processes and is considered a promising cancer drug target [53]. It also acts as a tumor suppressor, potentially through promoting the degradation of apoptosis inhibitor proteins [54]. Retinoic acid-related orphan receptor C (*rorc*) regulates cell proliferation, metastasis, and chemoresistance in multiple malignancies [55] and has predictive value for BC prognosis within certain limits [56]. These findings suggest that artemisinin may affect tamoxifen-resistant BC through mechanisms directly acting on its target genes, as also corroborated by PCR experiments.

Artemisinin interestingly targets well-known BC-associated genes such as *tp53, ar, erbb2*, and *esr1*, yet these were not included in the TAMGs. This might be because the LCC9 cell line and ER-positive BC tissues exhibit similar gene expression patterns, but these patterns do not reach a level of significance. Consequently, these crucial BC genes may not play a significant role in tamoxifen resistance. Nonetheless, the fact that artemisinin’s predicted targets include these key tumor genes suggests its potential for directly killing tumor cells. The challenge of reversing tumor cell resistance to established first-line drugs remains significant.

In using the MCODE plugin to identify highly interconnected regions in the PPI network, it was observed that not all intersected genes, except for *pgr, egfr*, and *mapk10*, were present in the core sub-clusters. However, both the PPI network and the three core sub-clusters showed a strong connection between artemisinin targets (green) and TAMGs (red). This implies that artemisinin might reverse tamoxifen resistance by directly regulating intersected TAMGs and indirectly through interactions between its target genes and TAMGs. Cluster 1 was identified as a key cluster. Correlation analysis between key-cluster genes and 22 types of immune cells was conducted in tamoxifen-sensitive and -resistant patients, respectively. Key-cluster genes were found to correlate significantly with different types of immune cells in each patient group. For example, in the tamoxifen-resistant patient group, B cells memory negatively correlated with genes like TP53, DNMT1, and positively with CDC25B, while in the tamoxifen-sensitive group, no genes showed significant correlation with B cells memory, but SOX2 was positively correlated with B cells naive. Evidence suggests that some genes and immune cells may have dual roles in tumor invasion and metastasis [57,58], indicating similar dual roles might exist in these key-cluster genes and immune cells. However, these findings could be influenced by the small sample size, lack of comprehensiveness, and non-representative nature of the samples in this study. Further research with a larger sample size is needed to validate these results.

ScRNA-seq analysis of 6 ER-positive BC samples highlighted the specific sensitivity of epithelial cells to artemisinin. Among these, cluster 3 exhibited notably higher sensitivity compared to other epithelial cell clusters, suggesting potential heterogeneity within BC cells. Tumor heterogeneity is a critical factor in the development of drug resistance due to variations among cancer cells [59]. Despite being annotated as epithelial cells, clusters 2, 3, 4, 7, 8, and 9 showed distinct molecular signatures. Precision medicine is therefore vital in treating drug-resistant cancer patients. Future research focusing intensively on cluster 3 could be pivotal, aiming to identify patients who are particularly sensitive to artemisinin.

The five genes with the most significant diverse expression levels among cell types are the androgen receptor (*ar*), cyclin-dependent kinase inhibitor 1A (*cdkn1a*), Erb-B2 receptor tyrosine kinase 2 (*erbb2*), estrogen receptor 1 (*esr1*), and heat shock protein 90 alpha family class A member 1 (*hsp90aa1*). Reliable evidence indicates that mutations or fusion proteins in *esr1* promote endocrine therapy resistance in ER-positive BC [60–62]. Interactions between *erbb2* and *er* can affect the growth and progression of ER-positive/HER2-positive BC [63]. The role of *ar* in ER-positive BC is debated, but recent evidence suggests *ar* may act as a tumor suppressor, making *ar*-targeted treatments promising [64,65]. CDKN1A, a tumor suppressor gene, when repressed, can facilitate BC cell proliferation and contribute to endocrine therapy failure [66]. HSP90AA1, identified as a cancer enabler, has been shown to be a critical factor in chemoresistance in osteosarcoma by regulating autophagy [67], with its high expression in BC often linked to poor prognosis [68].

The molecular docking results demonstrated that artemisinin effectively binds with the 7 intersected genes and 5 signature genes. This indicates that artemisinin may directly reverse tamoxifen resistance by binding with its target genes. Additionally, *in vitro* cell experiments confirmed its capability to reverse tamoxifen resistance in the LCC9 cell line by inhibiting cell proliferation and migration. PCR and WB analysis suggests that artemisinin may also influence resistance by regulating the expression of these target genes. It is noteworthy that, even after intervention with *ar*, the expression profiles of the drug-resistant LCC9 cell line show significant differences compared to the sensitive MCF-7 cell line. While the expression of 11 genes consistently remains significant, only the *ar* signature gene exhibits a closer similarity to the sensitive MCF-7 cell line. Research indicates that reducing the occurrence of tam resistance can be achieved by blocking the *ar* signaling pathway. AR plays an inhibitory role in estrogen-dependent diseases [69], and in approximately 90% of ER-positive breast cancers, *ar* shows positive expression, correlating with better patient prognosis [70]. The process of reversing resistance is achieved by increasing the expression of *ar* in resistant cell lines to levels closer to those in sensitive cell lines. Our research will focus on a comprehensive exploration of the mechanisms through which artemisinin reverses tamoxifen resistance by modulating the expression of the ar gene.

However, this study has limitations, including its preliminary focus on artemisinin’s effects exclusively *in vitro*. Future *in vivo* studies and clinical trials are necessary to comprehensively evaluate its efficacy and potential clinical applications. Despite these limitations, this research is pioneering in proposing the reversal effect of artemisinin on tamoxifen resistance, making it a unique contribution to the field.

## Conclusion

By integrating bioinformatics analysis with experimental validation, this study provides an initial examination of the interaction between artemisinin and tamoxifen at the genetic level. The findings highlight artemisinin’s potential to counter tamoxifen resistance in ER-positive breast cancer.

## Supplementary Materials

Figure S1. *p*-values of 1-20 PCs calculated by dimheatmap.

Figure S2. The resolution was set at 0.5 via clustree. A value of 18 emerged as the optimal PC, as it represents the last point where the change in percentage variation exceeds 0.1%.

Figure S3. Heatmap showing the top 5 significant marker genes (ar, cdkn1a, erbb2, esr1, hsp90aa1) for 18 single-cell clusters

Figure S4. Score hetmap showed that these 18 cell clusters were annotated into 7 cell types (T cells, B cells, epithelial cells, fibroblasts, tissue stem cells, endothelial cells and macrophage.

## Data Availability

The datasets analysed during the current study are available in GEO database (https://www.ncbi.nlm.nih.gov/geo/). The accession numbers of GEO datasets are GSE159968, GSE17705 and GSE161529.

## Abbreviations

BC: Breast cancer
ER: Estrogen receptor
HR: Hormone receptor
TAM: Tamoxifen
ART: Artemisinin
DO: Disease ontology
KEGG: Kyoto encyclopedia of genes and genomes
CYP2E1: Cytochrome P450 family 2 subfamily E member 1
RORC: Retinoic acid-related orphan receptor C
MAPK10: Mitogen-activated protein kinase 10
GLP1R: Glucagon like peptide 1 receptor
EGFR: Epidermal growth factor receptor
PGR: Progesterone receptor
MGLL: Monoglyceride lipase
AR: Androgen receptor
CDKN1A: Cyclin dependent kinase inhibitor 1A
ERBB2: Erb-B2 receptor tyrosine kinase 2
ESR1: Estrogen receptor 1
HSP90AA1: Heat shock protein 90 alpha family class A member 1
SERDs: Selective estrogen receptor down-regulators
AIs: Aromatase inhibitors
SERMs: Selective estrogen receptor modulators
TME: Tumor micro-environment

## Supplementary Materials

The supplementary material is available online at https://doi.org/10.32604/or.2024.047257.
