# Supplementary figures and images for: Reversal of tamoxifen resistance by artemisinin in ER+ breast cancer: bioinformatics analysis and experimental validation
Source: Oncol Res. 2024 May 23;32(6):1093–107. doi: 10.32604/or.2024.047257 (PMC11136689; doi:10.32604/or.2024.047257)

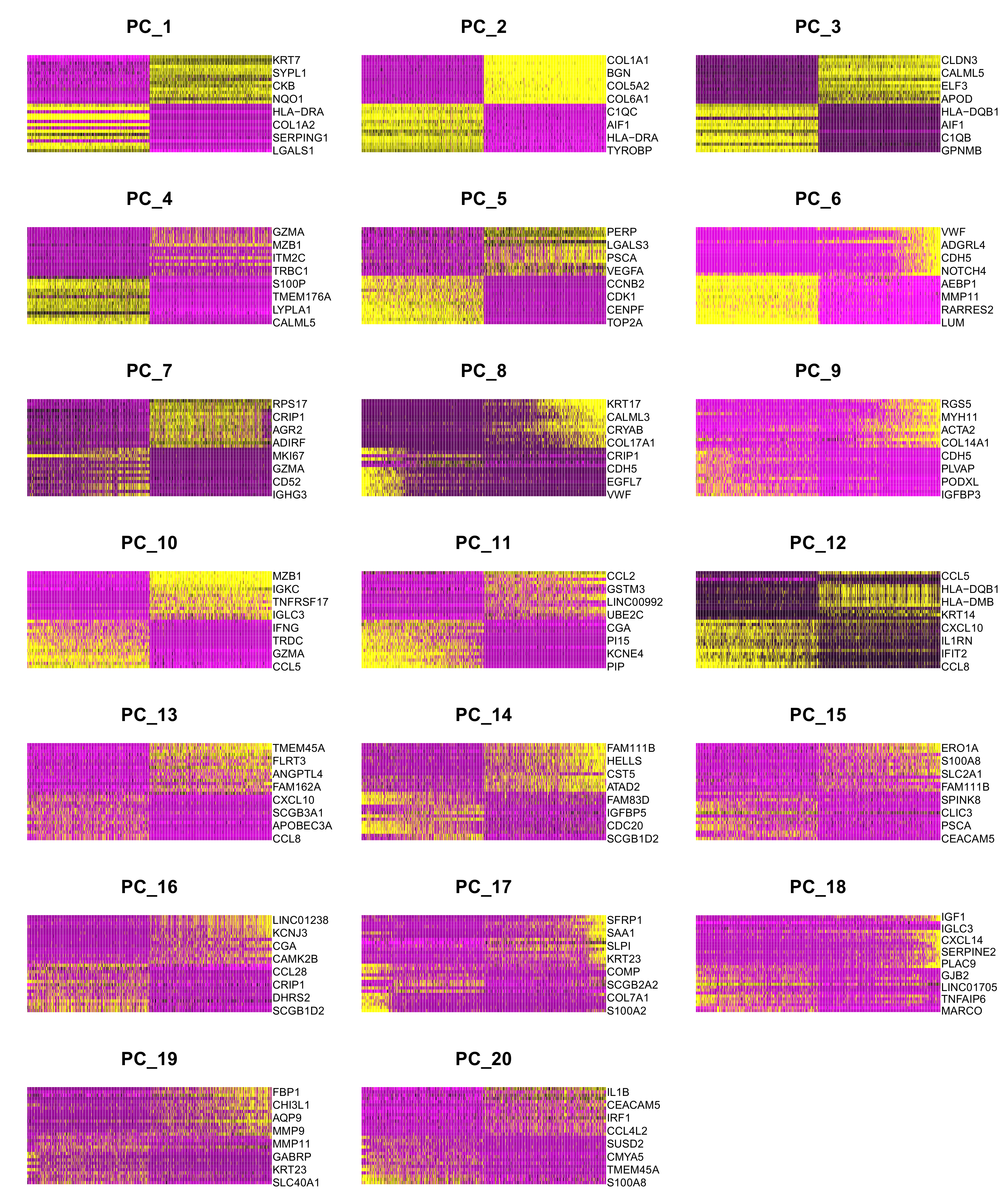

Supplement: Figure S1. [file OncolRes-32-47257-s001.tif]

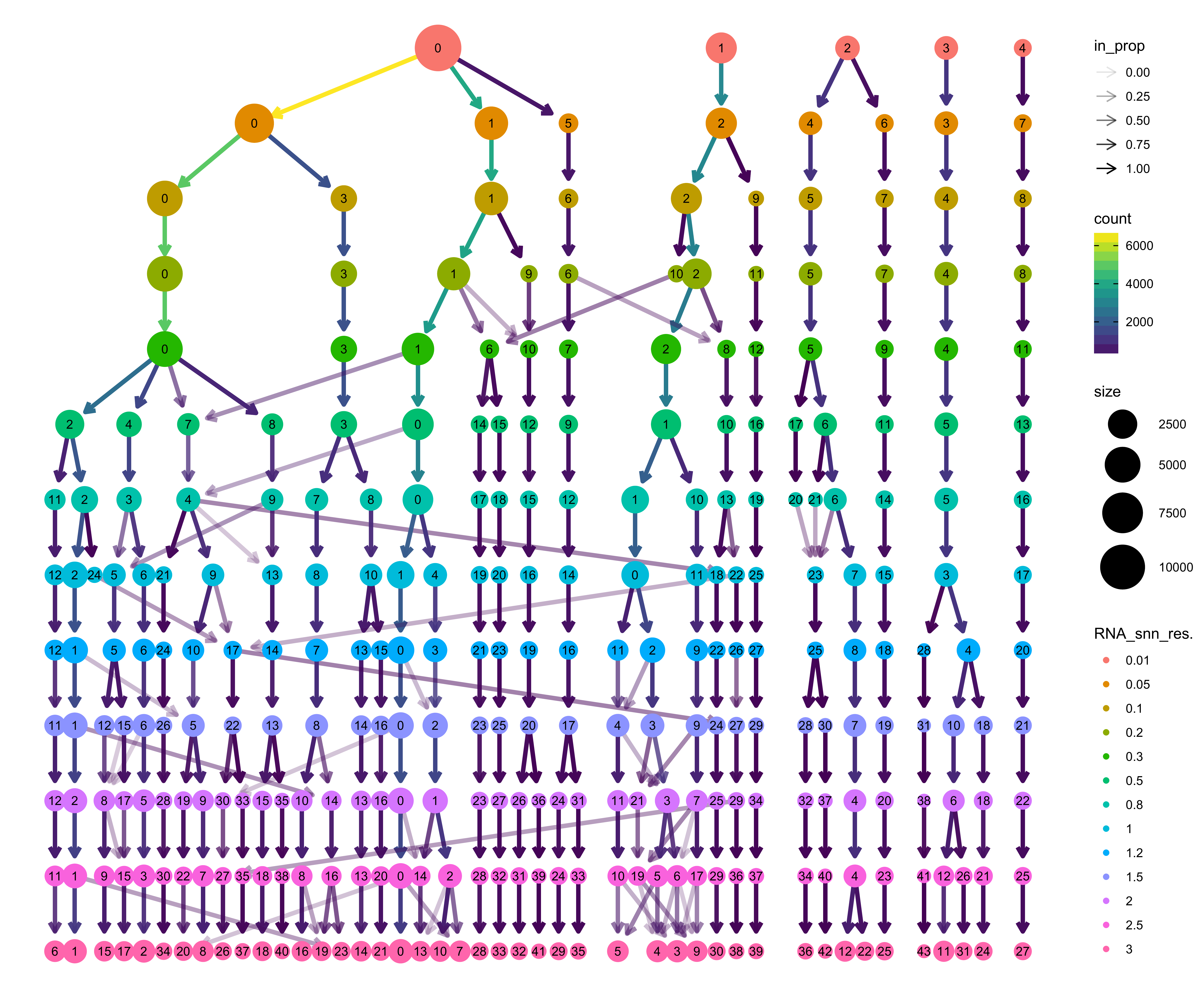

Supplement: Figure S2. [file OncolRes-32-47257-s002.tif]

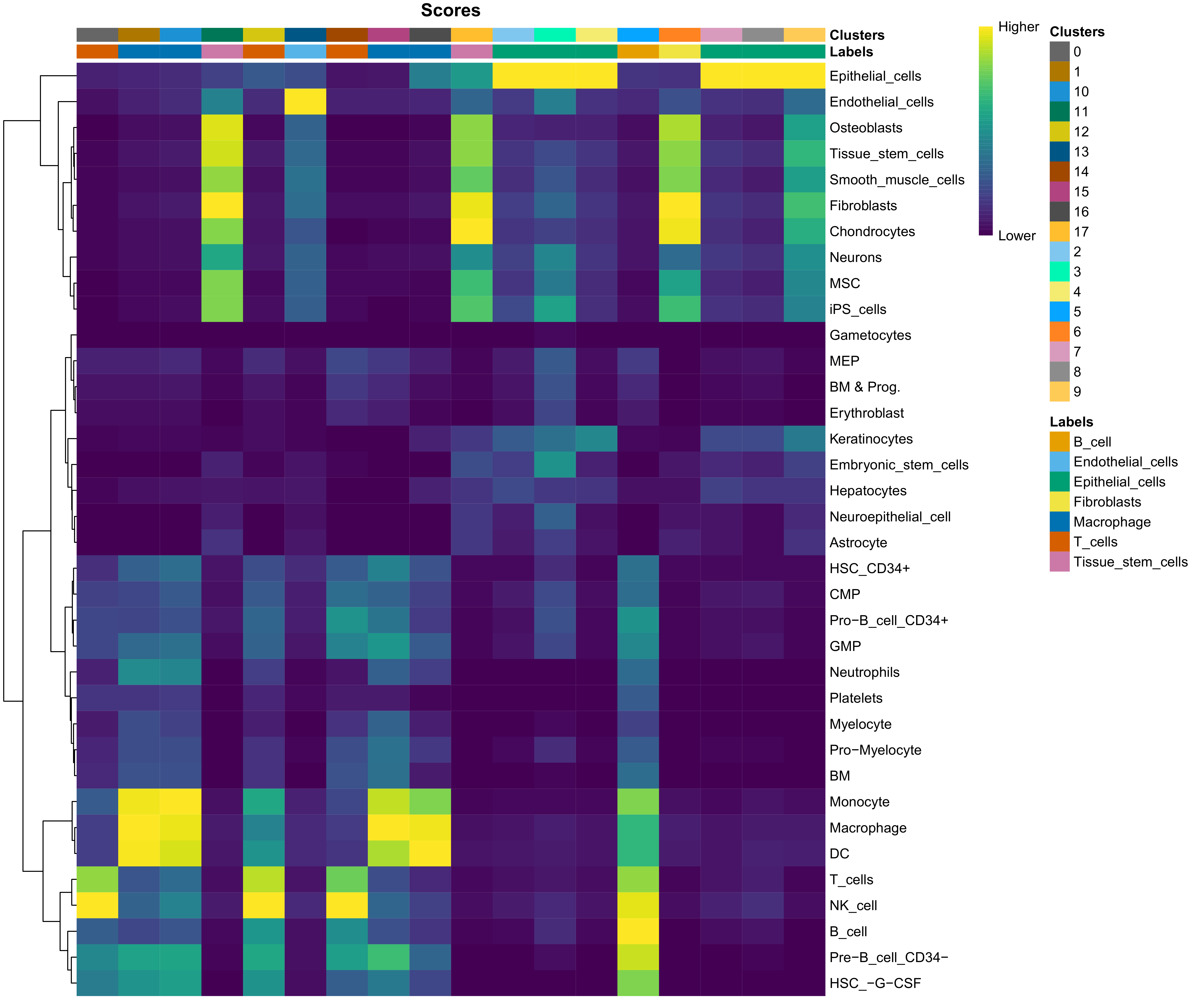

Supplement: Figure S4. [file OncolRes-32-47257-s004.tif]
